# A survey of carboplatin desensitization therapy in Japan: A multicenter retrospective study

**DOI:** 10.1002/cam4.6968

**Published:** 2024-03-16

**Authors:** Hiroaki Komatsu, Koji Matsumoto, Mitsunori Morita, Takayuki Nagasawa, Hiroshi Nishio, Jiro Suzuki, Shin Nishio, Hisanori Kobara, Mayu Yunokawa, Kazuya Ariyoshi, Takashi Hirayama, Hideki Tokunaga, Masayo Ukita, Kaori Yoriki, Mayuyo Mori‐Uchino, Akiko Furusawa, Shinichi Togami, Hiroko Nakamura, Mitsuya Ishikawa, Toyomi Satoh

**Affiliations:** ^1^ Department of Obstetrics and Gynecology Tottori University School of Medicine Yonago Tottori Japan; ^2^ Department of Medical Oncology Hyogo Cancer Center Akashi Hyogo Japan; ^3^ Department of Obstetrics and Gynecology Iwate Medical University School of Medicine Morioka Iwate Japan; ^4^ Department of Obstetrics and Gynecology Keio University School of Medicine Tokyo Japan; ^5^ Department of Obstetrics and Gynecology The Jikei University School of Medicine Tokyo Japan; ^6^ Department of Obstetrics and Gynecology Kurume University School of Medicine Kurume Japan; ^7^ Department of Obstetrics and Gynecology Shinshu University Nagano Japan; ^8^ Department of Gynecology The Cancer Institute Hospital of JFCR Tokyo Japan; ^9^ Department of Gynecology Service National Kyushu Cancer Center Fukuoka Japan; ^10^ Department of Obstetrics and Gynecology Juntendo University Hospital Tokyo Japan; ^11^ Department of Gynecology Tohoku University Graduate School of Medicine Sendai Japan; ^12^ Department of Gynecology and Obstetrics, Graduate School of Medicine Kyoto University Kyoto Japan; ^13^ Department of Obstetrics and Gynecology, Graduate School of Medical Science Kyoto Prefectural University of Medicine Kyoto Japan; ^14^ Department of Obstetrics and Gynecology, Faculty of Medicine The University of Tokyo Tokyo Japan; ^15^ Department of Gynecology Tokyo Metropolitan Cancer and Infectious Diseases Center, Komagome Hospital Tokyo Japan; ^16^ Department of Obstetrics and Gynecology, Faculty of Medicine Kagoshima University Kagoshima Japan; ^17^ Department of Obstetrics and Gynecology National Hospital Organization Kure Medical Center and Chugoku Cancer Center Kure Japan; ^18^ Department of Gynecology, National Cancer Center Hospital Tokyo Japan; ^19^ Department of Obstetrics and Gynecology, Institute of Medicine University of Tsukuba Tsukuba Japan

**Keywords:** carboplatin, desensitization, erythema, hypersensitivity reaction, pruritus

## Abstract

**Introduction:**

Hypersensitivity reactions (HSRs) to chemotherapy are serious adverse events associated with cancer drug therapy and can occur with any antitumor drug. This study investigated the safety and efficacy of carboplatin desensitization therapy in Japan and established a method for treating carboplatin HSRs.

**Methods:**

Patients diagnosed with gynecological (ovarian, endometrial, or cervical) cancers who underwent carboplatin desensitization therapy between 2016 and 2020 at the Gynecologic Cancer Study Group of Japan Clinical Oncology Group were included. The carboplatin desensitization therapy at each institution and the implementation cases were registered in an online case report form.

**Results:**

This retrospective study enrolled 136 patients (ovarian, 108; endometrial, 17; and cervical cancer, 11). Pre‐existing allergies were present in 37 (27.2%) patients, and 32 (23.5%) patients exhibited prodromal symptoms during treatment before HSR onset. Erythema was the most common symptom at HSR onset, affecting 93 (68.4%) patients, followed by itching in 72 (52.9%) patients and decreased oxygen saturation in 43 (31.6%) patients. Loss of consciousness occurred in three (2.2%) patients. The most common timing of HSR onset was during the first recurrence treatment (47%). The mean total carboplatin dose until HSR onset was 7331 (2620–18,282) mg, and the mean number of doses was 14 (4–63). Desensitization treatment was completed in 75% of cases, and breakthrough HSRs occurred in 25% (34/136). No deaths occurred in the study cohort. The risk factors for HSRs were not identified.

**Conclusion:**

Although carboplatin desensitization therapy has high success rates in Japan, erythema and pruritus are important HSRs to consider.

## Graphical abstract

Erythema was the most common symptom at HSR onset, affecting 93 (68.4%) patients, followed by itching in 72 (52.9%) patients and decreased oxygen saturation in 43 (31.6%) patients. Desensitization treatment was completed in 75% of cases, and breakthrough HSRs occurred in 25% (34/136).

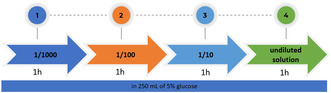

## 1 INTRODUCTION

The platinum and taxane combination regimen is the primary treatment method for both initial and recurrent cases of gynecologic cancers.^1^, ^2^, ^3^, ^4^, ^5^, ^6^, ^7^ Carboplatin, in particular, was introduced in 1989 as a treatment for ovarian cancer and has become a popular choice mainly because its therapeutic effect is comparable to that of cisplatin and it has fewer adverse events. However, hypersensitivity reactions (HSRs) have been reported in patients receiving multiple cycles of chemotherapy with carboplatin.^8^ The frequency of HSRs varies among different reports, and few detailed causes or risk factors have been reported.^9^ Previously, Altwerger et al.,^10^ Suggimoto et al.,^11^ and Joly et al.^12^ reported germline BRCA mutations, carboplatin dosage, and regimen type and age >70 years, respectively, as risk factors for HSRs. Although patients with a history of allergies are more susceptible to HSRs, the number of such cases is unknown.^13^, ^14^, ^15^

HSRs can severely impact vital signs, potentially leading to shock. Re‐administration of carboplatin after HSRs can lead to death.^16^ In addition, HSRs prevent the use of platinum drugs in patients with platinum‐sensitive relapsed disease despite the possibility of a successful platinum regimen. This may impact patients' prognoses. Carboplatin desensitization therapy has been used for HSRs in recent years, and several reports have been published.^17^, ^18^, ^19^, ^20^, ^21^, ^22^, ^23^, ^24^, ^25^, ^26^ However, no fixed method of administration has been established because the reports were from single centers, included a small number of cases, and used various methods. Furthermore, due to the complexity of the administration system at each facility, a certain method of administration has not been established.

This study aimed to investigate the therapy completion rate, frequency of breakthrough HSRs, and factors associated with their onset in patients treated with carboplatin desensitization therapy. We defined HSRs that occurred during the desensitization treatment period as breakthrough HSRs.

## 2 METHODS

Patients who underwent carboplatin desensitization therapy for gynecological (cervical, uterine body, primary ovarian, fallopian tube, and peritoneal) cancers at the participating centers of the Gynecologic Cancer Study Group of the Japanese Clinical Oncology Group (JCOG) between January 2016 and December 2020 were included. The indication for desensitization therapy is left at the discretion of the gynecologic or medical oncologist at each facility and approved for implementation. A patient record form created using Google Forms was sent to each facility, and the cases were registered online. Each facility was asked to enter a case number to verify the case details.

**FIGURE 1:**
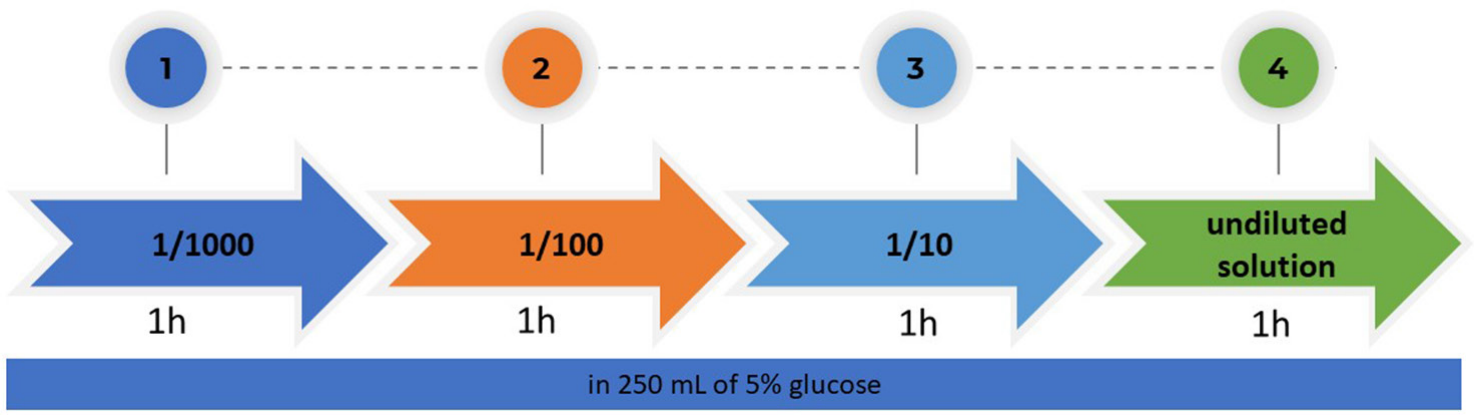
Typical scheme of carboplatin de‐sensitization. Solutions with 1/1000, 1/100, and 1/10 dilutions of carboplatin and an undiluted solution were prepared in 250 mL of 5% glucose.

For each facility, the carboplatin solution, administration rate, dilution method (e.g., 1/1000, 1/100, 1/10, or undiluted solution), and place of administration (including a private room, large room, intensive care unit, or outpatient clinic) were recorded. The typical treatment protocol was to administer the therapy for 1 h per session for 4 h (Figure 1).

The individual case questionnaire included details about age, type of cancer, FIGO stage, presence of allergies, HSR onset information, number of doses of the platinum regimen (including conventional, dose‐dense, or weekly regimen), total dose, presence of suspected symptoms at the time of treatment one cycle prior to the treatment in which HSRs were diagnosed, symptoms before HSR onset timing, symptoms, response to HSR onset, number of desensitization therapies administered, prophylactic agents, duration of administration, and HSR recurrence.

The primary endpoints were the completion rate of carboplatin desensitization therapy and the frequency of breakthrough HSRs in patients treated with desensitization therapy. Secondary endpoints were the development of carboplatin HSRs and their associated factors. Cases of complete treatment included those in which the planned desensitization therapy was administered or discontinued due to disease progression. Risk factors for the development of breakthrough HSRs were extracted using multivariate analysis.

This study was approved by the Ethics Committee of the Tottori University Hospital (IRB number 21‐018) and all participating facilities. The requirement for obtaining informed consent was waived owing to the retrospective nature of the study.

### 2.1 Statistical analyses

Continuous data are presented as mean and standard deviation. Categorical data are presented as numbers and frequencies. Multivariate analysis using logistic regression analysis was performed. Statistical significance was set at *p* values of <0.05. All statistical analyses were performed using the GraphPad Prism 8.3 software (GraphPad Software, Inc., La Jolla, CA, USA).

## 3 RESULTS

Twenty facilities participated in the study. A total of 136 patients (ovarian cancer, 108; endometrial cancer, 17; cervical cancer, 11) were enrolled in this retrospective study (Table 1). A total of 638 cycles of desensitizing therapy were administered. All centers used a four‐step method starting with a 1000‐fold dilution. Solutions with 1/1000, 1/100, and 1/10 dilutions of carboplatin and an undiluted solution were prepared in 250 mL of 5% glucose. Each solution was administered as a 1‐h intravenous infusion (4‐step 4‐h protocol).^18^ Sixteen facilities (80%) administered the therapy in a large room and 4 (20%) in an observation room. In 64% of the facilities, physicians were on call in the wards, 25% were on call at the bedside, and the remainder appeared to be called in the event of an emergency.

**TABLE 1:** Patient characteristics.

	Total (*n* = 136)	Ovarian (*n* = 108)	Endometrial (*n* = 17)	Cervical (*n* = 11)
Age (years)	54 (27–82)	54 (27–82)	56 (32–81)	45 (29–58)
Height (cm)	156 (132–169)	156 (132–168)	156 (145–162)	158 (152–169)
Body weight (kg)	54 (39–100)	51 (32–89)	62 (42–100)	58 (47–67)
Stage				
I	16	6	3	7
II	4	3	0	1
III	75	69	5	1
IV	39	28	9	2
Unknown	2	2	0	0
Carboplatin dosage regimen				
Conventional	99	72	16	11
Dose‐dense	31	30	1	0
Weekly	6	6	0	0
Allergy				
Yes	37 (27.2%)	32 (29.6%)	0	5 (45.5%)
No	99 (72.8%)	76 (70.4%)	17 (100%)	6 (54.5%)
Suspected symptoms of previous treatment				
Yes	32 (23.5%)	25 (23.1%)	3 (17.6%)	4 (36.4%)
No	104 (76.5%)	83 (76%)	14 (82.4%)	7 (63.6%)
Breakdown of suspected symptoms of previous treatment				
Erythema	19	14	2	3
Itching	18	13	2	3
Nausea and vomiting	5	5	0	0
Dyspnea	4	4	0	0
Other	5	4	1	0

Overall, 37 (27.2%) patients had preexisting allergies and 32 (23.5%) exhibited prodromal symptoms in the previous cycle when the HSR occurred. The most common symptoms were erythema and itching.

**FIGURE 2:**
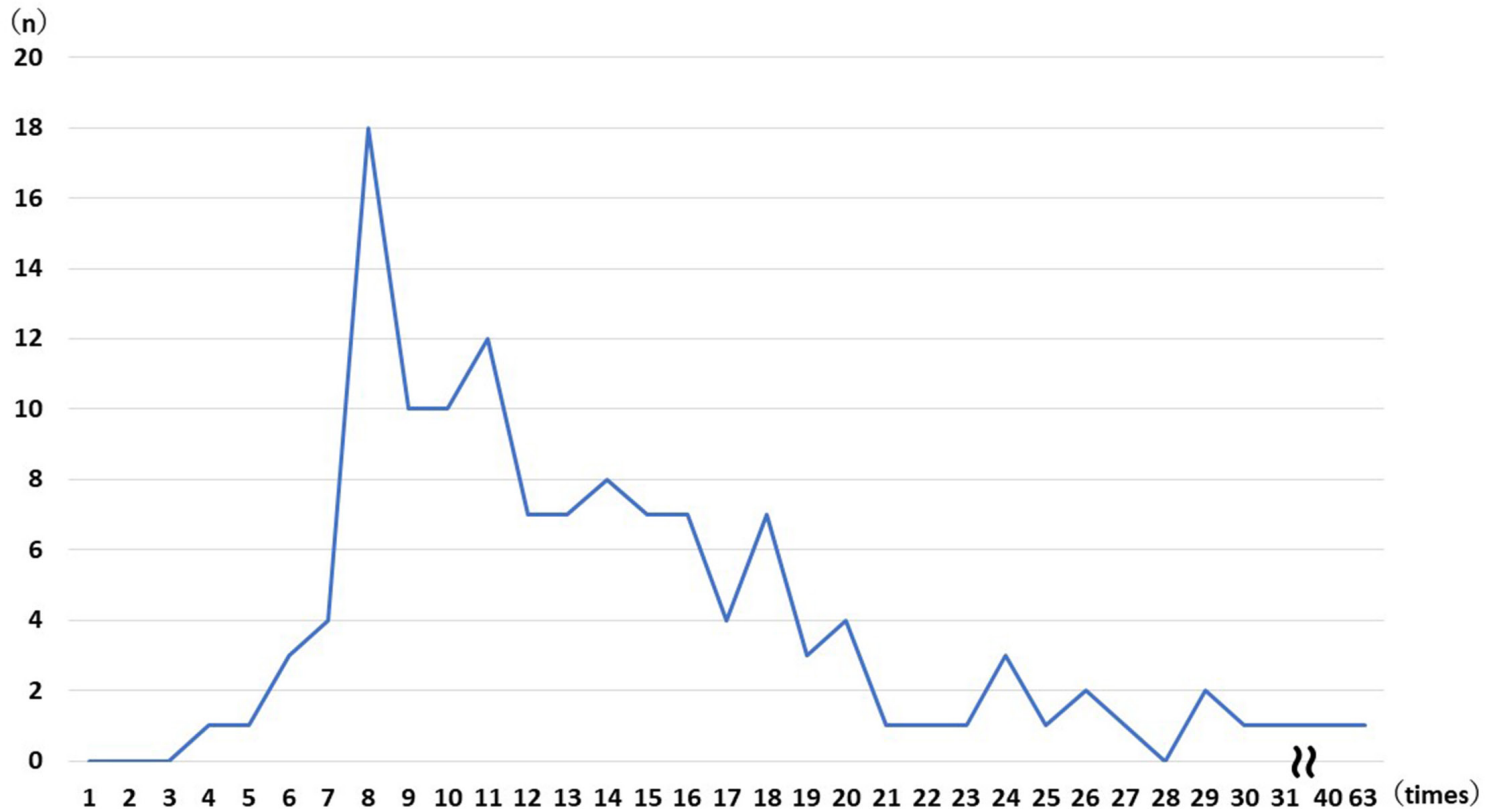
Number of carboplatin treatment courses until HSRs. HSRs occurred more frequently after the eighth cycle of treatment administration. HSRs, hypersensitivity reactions.

At HSR onset, erythema was the most common symptom, occurring in 93 (68.4%) patients, followed by itching in 72 (52.9%) and hypoxia in 43 (31.6%) patients (Table 2). Loss of consciousness occurred in three patients (2.2%). The timing of HSR onset was first‐line treatment in 31 (22.8%) patients, second‐line treatment in 63 (47.1%), and third‐line treatment or later in 41 (30.1%). The mean total carboplatin dose until HSRs appeared was 7331 (2620–18,282) mg, and the mean number of doses was 14 (4–63) (Table 3). HSRs occurred more frequently after the eighth cycle of treatment administration (Figure 2).

**TABLE 2:** Common symptoms at HSRs.

	Total (*n* = 136)	Ovarian (*n* = 108)	Endometrial (*n* = 17)	Cervical (*n* = 11)
Erythema				
All	93 (68.4%)	71	13	10
<10%	45	35	6	4
10%–30%	18	13	2	3
≥30%	16	10	4	2
Unknown	14	13	1	0
Itching				
Yes	72 (52.9%)	53	10	9
Sensory disturbance				
Yes	24 (17.6%)	23	1	0
Nausea and vomiting				
All	37 (27.2%)	31	3	3
Mild	31	25	3	3
Severe	4	4	0	0
Unknown	2	2	0	0
Dyspnea				
All	43 (31.6)	37	3	3
Mild (SpO_2_ 90–95%)	27	21	3	3
Moderate (SpO_2_ < 90%)	7	7	0	0
Severe	1	1	0	0
Unknown	8	8	0	0
Systolic blood pressure values				
91–100 mmHg	1	1	0	0
81–90 mmHg	7	7	0	0
71–80 mmHg	6	6	0	0
≥70 mmHg	4	4	0	0
Unknown	5	4	1	0
Chest discomfort				
Yes	24 (17.6%)	22	0	2
Unconsciousness				
Yes	3 (2.2%)	3	0	0

**TABLE 3:** Mean number of carboplatin doses and total number of carboplatin doses until HSR appearance.

	Total (136)	Ovarian (*n* = 108)	Endometrial (*n* = 17)	Cervical (*n* = 11)
Total carboplatin dose until HSR appearance (mg) average	7331 (2620–18,282)	7398 (2620–18,282)	7611 (4728–13,390)	5948 (2756–12,199)
Total number of carboplatin doses until HSR appearance (*n*) average	14 (4–63)	14 (4–63)	14 (8–18)	12 (8–21)

Abbreviation: HSR, Hypersensitivity reaction.

**FIGURE 3:**
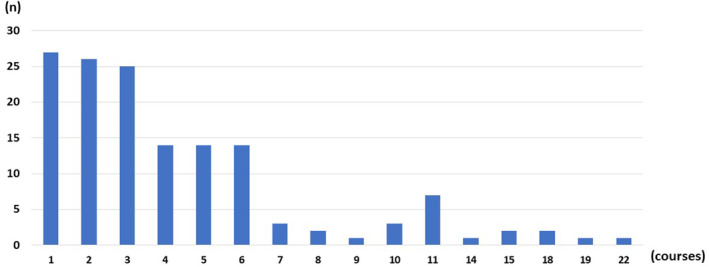
Number of carboplatin desensitization treatment courses. The median number of treatment courses was 3; some patients received up to 22 courses.

Desensitization therapy was completed in 75% of patients, with 25% (34/136) experiencing breakthrough HSRs (i.e., HSRs that occurred during the desensitization treatment period) (Table 4). Among these patients, 29% (10/34) of patients met all the diagnostic criteria for anaphylaxis.^21^ The median number of desensitization treatments was 3, with some patients receiving up to 22 courses (Figure 3). No HSR‐related mortality was observed. Risk factors for HSRs were not identified in this study (Table 5).

**TABLE 4:** Treatment complete rate.

	Total (136)	Ovarian (*n* = 108)	Endometrial (*n* = 17)	Cervical (*n* = 11)
Treatment complete rates	102 (75.0%)	83 (76.9%)	14 (82.4%)	5 (45.5%)
Reason for discontinuation				
Progression of disease	30 (20.1%)	23 (18.8%)	4 (23.5%)	3 (28.3%)
Breakthrough HSRs	34 (25.0%)	25 (33.7%)	3 (17.6%)	6 (63.6%)
Patient wishes	6 (4.4%)	3 (2.8%)	3 (17.6%)	0
Others	4 (2.9%)	3 (2.8%)	1 (5.9%)	0

Abbreviation: HSRs, Hypersensitivity reactions.

**TABLE 5:** Risk factors for HSRs.

	Odds ratio	95% CI	*p*‐value
Age (54 years)	1.263	0.478–3.424	ns
Allergy (drugs)	1.219	0.349–3.793	ns
Suspected symptoms of previous treatment	0.462	0.099–1.579	ns
NAC‐IDS or PDS	0.557	0.204–1.529	ns
Timing of treatment	0.932	0.310–3.303	ns

## 4 DISCUSSION

Our study is one of the largest studies on carboplatin desensitization. This study showed that the main symptoms at HSR onset were erythema and itching. The completion rate was 75%, indicating that the four‐step carboplatin desensitization regimen was safe and feasible. However, the risk factors for breakthrough HSR development could not be determined.

Regarding symptoms preceding HSR diagnosis, erythema and itching, which can be considered prodromal symptoms, were observed in approximately 20% of patients during the course of treatment before HSR onset. However, in some centers, these symptoms could be considered HSRs, making it challenging to distinguish between them. In this study, the symptoms spontaneously resolved, and carboplatin therapy was continued even after symptom onset because the symptoms were not considered severe. Based on these results, we believe that early diagnosis of HSRs may be difficult, especially when only accompanied by atypical prodromal symptoms.

At HSR onset, erythema followed by itching appeared as a prodromal symptom. Erythema usually occurs in less than 10% of the body, and the appearance of symptoms may be missed owing to clothing. Altwerger et al.^16^ reported that the frequency of erythema on the palms was 30.3%, which was lower than that observed in our study; however, no generalized erythema was reported. They also reported pruritis in 51.5% of their patients, which was comparable with the results observed in the present study. Owing to the presence of hypoxia and hypotension as symptoms other than erythema, desensitization should be performed in an environment where epinephrine can be administered immediately. In this study, we also examined the location and system of desensitization administration and conducted a subgroup analysis.

The mean total dose of carboplatin at HSR onset was approximately 7000 mg, and the mean number of courses was 14. These were similar regardless of carcinoma type and were predictors of HSR onset when carboplatin combination therapy was used. It should be noted that the earliest symptoms of HSR onset occurred in the fourth course, and the latest occurred in the 63rd course; thus, they may still occur after long‐term treatment.

The treatment completion rate for desensitization administration was 75%, which was lower than that reported by another study. Yamamoto et al.^25^ reported a treatment completion rate of 93.4%, which can vary greatly depending on the definition of treatment completion. The primary endpoint of their study was the success rate of carboplatin desensitization administration, which was calculated as the percentage of patients who completed all planned cycles among all patients and the percentage of completed carboplatin desensitization cycles out of the total number of cycles. However, our treatment completion rate is the percentage of patients who completed treatment without breakthrough HSRs, which may explain the difference. The differences in cancer type, small number of cervical cancer cases, and response rate to platinum as a treatment for recurrence may have had an impact. Breakthrough HSRs were observed in 25% of patients, which was comparable to that reported by Altwerger et al. (14/56). We did not analyze the details of the occurrence of breakthrough HSRs in this study. We would like to address this in future studies. The presence or absence of allergy was not reliably ascertained. Because a drug or food allergy does not manifest unless ingested, it is not possible to reliably identify whether the patient has a true allergy.^26^

Risk factors for HSRs could not be identified. Although the frequency of allergy varies with drug interactions, the findings of Joly et al.^26^ are worth noting. HSR onsets may be influenced by the individual's allergic constitution and the frequency of prior supportive care, such as antihistamines and steroids. Future studies should further investigate these factors.

### 4.1 Limitations and strengths

This study had some limitations. First, this was a retrospective study and was prone to various biases. We are currently planning to conduct a prospective study and look forward to future research in this area. Second, the diagnostic criteria for HSRs have not been clearly established. It would be desirable to have a research plan that clearly defines the frequency of occurrence based on diagnostic criteria for anaphylaxis or the measurement of tryptase levels at the event. Current CTCAE (ver.5) has three possible terms refuting HSR, including “allergic reaction,” “infusion‐related reaction,” and “anaphylaxis.” “Allergic reaction” and “infusion‐related reaction” are graded according to the intervention used for their management. “Anaphylaxis” is only classified as grade 3 or 4. Therefore, relatively mild but potentially dangerous reactions are difficult to grade using the CTCAE. The World Allergy Organization (WAO) Anaphylaxis Guidelines require skin (and/or mucosa) symptoms plus at least one other systemic symptom for the diagnosis of anaphylaxis. Consequently, if a patient experiences only generalized urticaria during carboplatin infusion, a diagnosis of anaphylaxis cannot be made using the WAO criteria. Usually, such patients are treated as those with HSR. This is also why only 29% of the patients who received desensitization fulfilled the anaphylaxis criteria. Third, the multicenter approach of this study was a limitation because of variations in desensitization protocols across institutions.

One of the strengths of this study is that it is one of the largest case analyses to date and includes more than 100 reported cases.

## 5 CONCLUSION

This study revealed that more than 20% of patients had prodromal symptoms when carboplatin was administered before one of the treatments, leading to an HSR diagnosis. Additionally, the main symptoms of carboplatin treatment HSR onset were erythema and itching. The completion rate of carboplatin desensitization therapy using the four‐step method was 75%, indicating that the regimen was safe and feasible, even for patients with HSRs. We are preparing to conduct a prospective multi‐institutional study on carboplatin desensitization in Japan.

## AUTHOR CONTRIBUTIONS

**Hiroaki Komatsu:** Data curation (equal); project administration (equal); writing – original draft (lead); writing – review and editing (equal). **Koji Matsumoto:** Conceptualization (equal); data curation (equal); supervision (equal); writing – review and editing (equal). **Mitsunori Morita:** Data curation (equal); writing – review and editing (equal). **Takayuki Nagasawa:** Data curation (equal). **Hiroshi Nishio:** Data curation (equal); writing – review and editing (equal). **Jiro Suzuki:** Data curation (equal); writing – review and editing (equal). **Shin Nishio:** Data curation (equal); supervision (equal). **Hisanori Kobara:** Conceptualization (equal). **Mayu Yunokawa:** Data curation (equal). **Kazuya Ariyoshi:** Data curation (equal). **Takashi Hirayama:** Data curation (equal). **Hideki Tokunaga:** Data curation (equal). **Masayo Ukita:** Data curation (equal). **Kaori Yoriki:** Data curation (equal). **Mayuyo Mori‐Uchino:** Data curation (equal). **Akiko Furusawa:** Data curation (equal). **Shinichi Togami:** Data curation (equal). **hiroko nakamura:** Data curation (equal). **Mitsuya Ishikawa:** Data curation (equal); supervision (equal); writing – review and editing (equal). **Toyomi Satoh:** Supervision (equal); writing – review and editing (equal).

## CONFLICT OF INTEREST STATEMENT

The authors have no conflict of interest.

## FUNDING INFORMATION

This study received no funding.

## ETHICS APPROVAL STATEMENT

The study was approved by the Ethics Committee of Tottori University (IRB number: 21‐018).

## PATIENT CONSENT STATEMENT

The requirement for obtaining informed consent was waived owing to the retrospective nature of the study. Patient consent was obtained by opt‐out.

## Data Availability

The data that support the findings of this study are available from the corresponding author upon reasonable request.
